# Geometric Phase in Twisted Topological Complementary Pair

**DOI:** 10.1002/advs.202304992

**Published:** 2023-09-22

**Authors:** Kun Zhang, Xiao Li, Daxing Dong, Ming Xue, Wen‐Long You, Youwen Liu, Lei Gao, Jian‐Hua Jiang, Huanyang Chen, Yadong Xu, Yangyang Fu

**Affiliations:** ^1^ College of Physics Key Laboratory of Aerospace Information Materials and Physics (MIIT) Nanjing University of Aeronautics and Astronautics (NUAA) Nanjing 211106 China; ^2^ School of Physical Science and Technology Jiangsu Key Laboratory of Thin Films Soochow University Suzhou 215006 China; ^3^ Department of Physics Xiamen University Xiamen 361005 China; ^4^ State Key Laboratory of Mechanics and Control for Aerospace Structures Nanjing University of Aeronautics and Astronautics (NUAA) Nanjing 211106 China

**Keywords:** geometric phase, orbital angular momentum, subwavelength acoustics, topological complementary pair, tunable wave control

## Abstract

Geometric phase enabled by spin‐orbit coupling has attracted enormous interest in optics over the past few decades. However, it is only applicable to circularly‐polarized light and encounters substantial challenges when applied to wave fields lacking the intrinsic spin degree of freedom. Here, a new paradigm is presented for achieving geometric phase by elucidating the concept of topological complementary pair (TCP), which arises from the combination of two compact phase elements possessing opposite intrinsic topological charge. Twisting the TCP leads to the generation of a linearly‐varying geometric phase of arbitrary order, which is quantified by the intrinsic topological charge. Notably distinct from the conventional spin‐orbit coupling‐based theories, the proposed geometric phase is the direct result of the cyclic evolution of orbital‐angular‐momentum transformation in mode space, thereby exhibiting universality across classical wave systems. As a proof of concept, the existence of this geometric phase is experimentally demonstrated using scalar acoustic waves, showcasing the remarkable ability in the precise manipulation of acoustic waves at subwavelength scales. These findings engender a fresh understanding of wave‐matter interaction in compact structures and establish a promising platform for exploring geometric phase, offering significant opportunities for diverse applications in wave systems.

## Graphical abstract

Geometric phase is a powerful strategy for controlling light waves, which is solely applicable to spin light and not available for wave fields lacking intrinsic spin. By utilizing the cyclic evolution of OAM transformation, this study presents a general approach through topological complementary pair to achieve geometric phase and showcases its remarkable proficiency in controlling sound waves at subwavelength scales.

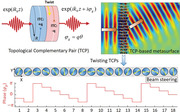

## 1 Introduction

Geometric phase is a universal concept in classic and quantum physics,^[^ ^1^, ^2^, ^3^, ^4^ ^]^ which refers to a phase factor acquired by a physical system as it undergoes a cyclic evolution in parameter space. The pioneering discovery of geometric phase was originally attributed to Pancharatnam in 1956, where he first noted a phase shift (now recognized as the Pancharatnam‐Berry geometric phase) arising from the introduction of cyclic changes within polarization space.^[^ ^5^ ^]^ When incident circularly‐polarized (spin) light propagates through an anisotropic material, it gets a space‐varying geometric phase of φ_*g*_ =  2θ, which is twice the orientation angle (θ) of the local optical axis of the material. As a novel phase control mechanism, the Pancharatnam‐Berry geometric phase has provided unprecedented opportunities for controlling freely light waves, particularly in the state‐of‐the‐art platforms offered by metasurfaces,^[^ ^6^, ^7^, ^8^ ^]^ leading to many promising applications in optics, including wavefront shaping,^[^ ^9^, ^10^ ^]^ vortex generator,^[^ ^11^ ^]^ flat metalens,^[^ ^12^, ^13^ ^]^ and holographic imaging,^[^ ^14^, ^15^ ^]^ to name a few. Despite these great achievements, these reported results primarily apply to circularlypolarized light and are greatly challenged by the simplest linearly‐polarized light and other structured fields. The fundamental reason is that the Pancharatnam‐Berry geometric phase arises from the spin‐orbit coupling of photons in anisotropic media. Moreover, this geometric phase is constrained to vector fields and cannot be extended to scalar fields without intrinsic spin (e.g., sound waves^[^ ^16^ ^]^), so it is not a general principle. Although some works have been reported to achieve geometric phases in scalar field systems via various mechanisms,^[^ ^17^, ^18^, ^19^, ^20^ ^]^ e.g., transverse spin, they are limited to specific values and do not span the complete phase range of 2π. Consequently, there is a pressing need to identify a mechanism that can realize geometric phase independent of intrinsic spin, which may open new avenues for controlling classical waves and broadening the scope of applications.

In contrast to spin angular momentum, orbital angular momentum (OAM), defined by a helical wavefront exp (*il*ϕ) (the integer *l* is topological charge), is not exclusive to light waves^[^ ^21^ ^]^ and it can also be manifested in other dynamic waves,^[^ ^22^, ^23^ ^]^ e.g., acoustic waves and elastic waves. Researchers have extensively studied the generation and conversion of OAM waves by designing bulky gradient structures^[^ ^24^ ^]^ and phase gradient metasurfaces (PGMs)^[^ ^25^, ^26^, ^27^, ^28^ ^]^ that possess intrinsic topological charge (ITC), leading to significant advancements in understanding and manipulating OAM across different wave modalities. An important advantage of OAM is its ability to exist in unbounded states, surpassing the two‐state limit of intrinsic spin, and it has resulted in significant applications across diverse scientific and technological domains,^[^ ^29^, ^30^, ^31^ ^]^ such as high‐capacity communications and molecule detection. These unique features of OAM make it an excellent candidate for exploring a universal principle behind the realization of geometric phase in classical wave systems, offering exciting possibilities for advancements in optics (e.g., high‐order geometric phase^[^ ^32^, ^33^ ^]^) and other wave‐based technologies.

In this work, we present both theoretically and experimentally a new and general approach for achieving geometric phase by introducing the concept of topological complementary pair (TCP). In analogy with complementary media^[^ ^34^, ^35^ ^]^ in transformation optics, which involve two adjacent slabs with opposite signs in material parameters, the proposed TCP is realized by two identical but complementary layers with ITC, i.e., each layer carrying a nonzero ITC but opposite to each other. This TCP design offers a feasible and efficient platform to manipulate OAM modes. Twisting the TCP can lead to a linearly‐varying geometric phase of arbitrary order, which is quantified by the ITC. This geometric phase is a direct result of the cyclic evolution of OAM transformation in mode space, which can be applied to classical wave systems including scalar fields. To illustrate these discoveries, we have devised experimental demonstrations of the TCP concept within acoustic waveguides, achieving the geometric phase at subwavelength dimensions. Leveraging the inherent advantages of the geometric phase, we showcase the efficacy of TCP‐based acoustic metasurfaces in freely manipulating acoustic planar and vortex fields, which offers a new way for designing tunable acoustic metasurfaces that goes beyond current limitations associated with complex passive methods^[^ ^36^, ^37^ ^]^ and expensive active techniques.^[^ ^38^, ^39^ ^]^ Our findings establish a highly promising foundation for exploring the concept of geometric phase in wave systems, and open new avenues for developing subwavelength acoustics,^[^ ^40^ ^]^ which may find important applications in wavefront control, OAM communications, holography imaging, and acoustic tweezers.

## 2 Results

### 2.1 Models and Theory for Geometric Phase Enabled by TCP

**Figure 1:**
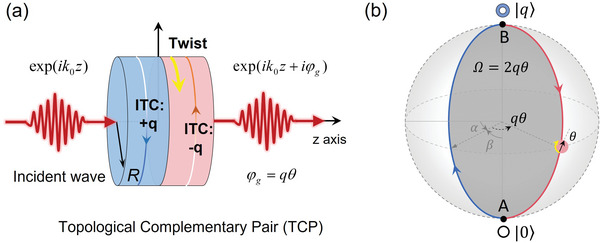
a) Schematic diagram of geometric phase in the twisted TCP that is achieved by two compact phase elements with opposite ITC. b) Bloch sphere for mode transformation between the fundamental plane mode (|0〉) and the OAM mode (|*q*〉) through the twisted TCP.

Let us start from the schematic diagram of the proposed TCP structure in **Figure** **1a**. The TCP is composed of two elements with identical structural configurations, and they are placed adjacent to one another. These two elements are designed with an antiparallel phase gradient in the azimuthal direction to introduce opposite ITC (*q*) for implementing the TCP. To be exact, the two elements carrying nonzero ITCs of + *q* (e.g., the left one) and − *q* (e.g., the right one) are complementary to each other, and governed by time‐reversal symmetry. Considering a plane wave of exp(*ik*_0_ *z*) (i.e., *q*  =  0) incident on such a twisted TCP structure from the left side, the transmitted wave is also a plane wave as the total topological charge of the TCP is zero. By twisting the TCP, e.g., rotating the right element of the TCP with an angle of θ, apart from the propagation phase accumulated across the TCP, the transmitted wave undergoes an additional geometric phase of exp(*ik*_0_ *z* + *i*φ_*g*_) with φ_*g*_ =  *q*θ.

The underlying physics originates from mode transformation between the two orthogonal OAM modes. The geometric phase can be understood using a unitary rotation in the two‐mode vector space and analyzed from the Bloch sphere (see Figure 1b) via the SU(2)‐SO(3) homomorphism.^[^ ^3^ ^]^ The trivial incident plane wave is denoted as |0〉, and it is converted into the OAM mode, denoted as |*q*〉, after passing through the left part of the TCP. Then the Bloch vector |**n**〉 is given by,

(1) $$|n\rangle =e^{-i\alpha /2}\left(\cos\frac{\beta }{2}\;|0\rangle +\sin\frac{\beta }{2}e^{i\alpha }|q\right\rangle )$$

where *β* and *α* are the polar and azimuthal angles in Bloch sphere. Note that two arbitrary orthogonal modes could be involved in Equation 1 to construct the Bloch sphere for studying the two‐state evolution and the related geometric phase. For the input mode |0〉 = (1,  0)^*T*^  located at the south pole (β  =  0) of the Bloch sphere, it could be converted to |**n**〉 by the unitary matrix,

(2) $$R\;\left(\beta ,\alpha \right)=\left(\begin{array}{cc} \cos\frac{\beta }{2}e^{-i\alpha /2}\; & -\sin\frac{\beta }{2}e^{-i\alpha /2}\\ \sin\frac{\beta }{2}e^{i\alpha /2} & \cos\frac{\beta }{2}e^{i\alpha /2} \end{array}\right)$$

The  + *q* element of the TCP works as a π‐converter operation with a rotation matrix *R*(π) ≡ *R*(β,  0), which transforms the input mode to the north pole of Bloch sphere, |*q*〉 =  *R*(π) |0〉 = (0,  1)^*T*^, as shown by the *A* → *B* path in Figure 1b. Immediately, the − *q* element provides a reverse π‐converter for the OAM mode |*q*〉, accompanied by a rotation operation, i.e.,

(3) $$T\;\left(\theta \right)=\left(\begin{array}{cc} 1 & 0\\ 0 & e^{iq\theta } \end{array}\right)$$

where θ is the twisting angle of the − *q* element, indicated by the yellow arrow on the red circle. This operation returns the OAM mode |*q*〉 to the mode |0〉, as shown by the *B* → *A* path. The whole process could be understood as a product of mode transformation,

(4) $$R\left(-\pi \right)T\left(\theta \right)R\left(\pi \right)\;|0\rangle =e^{iq\theta }\;\left|0\right\rangle$$

which implies that the twisted TCP can transform the input mode back to itself and offers an additional phase factor *e* ^*iq*θ^. In analogy to the Pancharatnam‐Berry geometric phase in optics, this phase is a geometrical phase given by φ_*g*_ =  Ω/2, where Ω is the solid angle enclosed by the *A* → *B* → *A* path in Figure 1b. This predicts the geometrical phase,

(5) $$\varphi_{g}=\;q\theta$$

Based on Equation 5, we find that twisting the TCP can produce a linearly‐varying geometric phase of arbitrary order, which is quantified by the ITC. This geometric phase can provide a new method for realizing high‐order geometric phase,^[^ ^32^, ^33^ ^]^ previously accomplished using nonlinear harmonic waves or rotationally‐symmetric structures in linear optics.

### 2.2 Robust Generation of Geometric Phase in Acoustics

**Figure 2:**
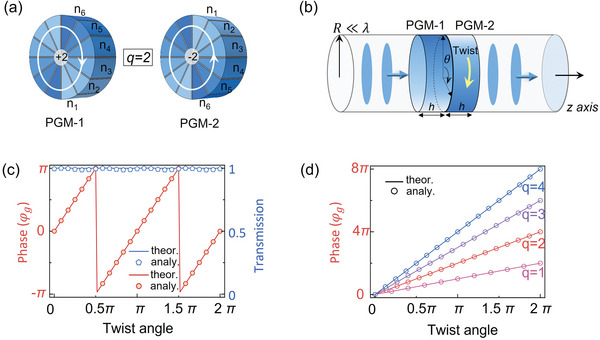
Theory model for realizing subwavelength TCP in acoustics. a) Subwavelength TCP enabled by a pair of PGMs (*q*  =  2) with a thickness of *h*. b) Acoustic waveguide structure with the twisted TCP. c) Geometric phase and transmission of the output wave through the twisted TCP with *q*  =  2 in theory (lines) and in analytical calculations (symbols). d) Geometric phase in twisted subwavelength TCPs with *q*  =  1,  2,  3,  4 in the acoustic waveguide.

We employ gradient index plates and spiral phase plates [see Text S1, Supporting Information] to design TCPs with ITC of *q*  =  1,  2,  3, and the geometric phase is numerically verified by scalar acoustic waves and linearly‐polarized light. Limited by the Rayleigh diffraction effect,^[^ ^41^ ^]^ the dimension of the TCP is generally large enough to ensure the availability of ITC, which is critical to achieving the proposed geometric phase. Fortunately, the dimensional limitation can be overcome by incorporating the concept of TCP in the design of acoustic cylindrical waveguides. Unlike other waveguide systems, acoustic waves do not experience a cutoff guided mode, allowing for greater flexibility of the TCP in a smaller dimension, including subwavelength scales. For example, we can realize the subwavelength TCP in airborne acoustics by employing a pair of PGMs,^[^ ^28^ ^]^ which are realized by *q* groups of fanlike supercells whose azimuthal phase distribution covers 2π discretized by *m* fanlike cells with thickness of *h* (see **Figure** **2a**). This azimuthal phase distribution in theory could be achieved by filling the gradient index profile *n_j_* =  1 + (*j* − 1)λ/*mh* in these fanlike waveguides (gray areas), where *j*  =  1,  2 ⋅⋅⋅*m*. In contrast with that in the PGM‐1, the gradient index profile in the PGM‐2 is placed with opposite angular distribution, as indicated by gradient colors in Figure 2a, so that they provide ITCs of + *q* and − *q*, respectively. By arranging this subwavelength TCP in the cylindrical waveguide (see Figure 2b), we show that twisting the PGM‐2 with an angle θ can lead to the robust generation of acoustic geometric phase. Although it is the evanescent OAM mode in the subwavelength waveguide, the cyclic evolution of mode transformation still works. To clarify this, we develop the coupled mode theory to analytically study the geometric phase in the acoustic waveguide [see Text S2, Supporting Information]. For example, Figure 2a displays the TCP with *q*  =  2, where six (*m*  =  6) cells are considered to design the supercell. As predicted by Equation 4, twisting the PGM‐2 allows the phase shift of the outgoing plane wave to have a linear dependence of φ_*g*_ =  2θ, and meanwhile, the transmission efficiency is unity independent of the twist angle. By considering *R*  =  0.15λ and *h*  =  0.45λ for the subwavelength PGMs to design the TCP, Figure 2c displays the analytical calculations (symbols), which agree with theoretical results (lines). Moreover, these TCPs with *q*  =  1,  2,  3,  4 are further used to demonstrate the robust generation of acoustic geometric phase at subwavelength scales (see Figure 2d), and they can work with a unity efficiency in all cases [see Text S3, Supporting Information]. Therefore, by controlling the ITC within the TCP, the proposed geometric phase of arbitrary order can be achieved.

### 2.3 TCP Design

**Figure 3:**
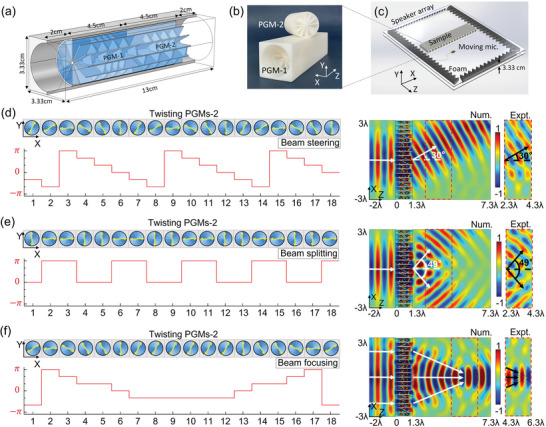
TCP design and experimental demonstration for tunable acoustic wave manipulation. a) Topography of the designed TCP made of space‐coiling metamaterials. b) TCP sample, where the PGM‐2 is fabricated individually for the twist operation. c) Experimental setup for measuring acoustic field patterns through the acoustic metasurface made of 18 TCP structures. Numerical and experimental demonstrations (right pixels) for the acoustic metasurface with beam steering d), splitting e), and focusing f) effects by twisting 18 PGMs‐2 with different orientations (yellow arrows) to achieve the required geometric phase profiles (left pixels), respectively. The experimentally measured areas are indicated by the corresponding dashed frames in simulations. The measured fields are normalized to the amplitude of the incident Gaussian beam in an empty waveguide.

The geometric phase enabled by the subwavelength TCP can provide a new way to manipulate acoustic fields in a tunable manner that goes beyond conventional limitations of propagation phase and resonant phase.^[^ ^36^, ^37^ ^]^ The TCP structure with *q*  =  2 in Figure 2a is used to demonstrate this claim. The PGMs are designed with *R*  =  1.5 cm, *h*  =  4.5 cm, and the working frequency is λ  =  10 cm. We use space‐coiling metamaterials^[^ ^28^ ^]^ to design PGMs and implement the TCP (see **Figure** **3a**) [see Text S4, Supporting Information]. To control acoustic waves in a planar geometry, the cylindrical waveguide is wrapped by a square cross‐section block with length λ/3  =  3.33 cm, which is slightly larger than the waveguide diameter (3 cm). Two matching layers (empty waveguides) with a length of 2 cm are added on both sides of the TCP structure (see Figure 3a) to obtain the geometric phase with perfect efficiency. By twisting the PGM‐2, the phase shift of the outgoing wave through the designed TCP has a linear dependence of φ_*g*_ =  2θ and its transmission efficiency can reach unity [see Text S4, Supporting Information], which is consistent with the ideal case in Figure 2c. Using 3D printing with commercial photopolymer materials, the TCP is fabricated and shown in Figure 3b, where the PGM‐1 is fixed and the PGM‐2 is fabricated individually for the twist operation.

### 2.4 Tunable Manipulation of Acoustic Waves at Subwavelength Scales

An acoustic metasurface composed of 18 TCP structures is implemented to demonstrate the tunable manipulation of acoustic plane waves. Experiments are performed in a parallel‐plate waveguide (1.2 × 1.2 m) with a gap of 3.33 cm (see Figure 3c), where a speaker array is used to generate a normally incident Gaussian beam and a moving microphone is used to measure acoustic pressure fields [see Text S5, Supporting Information]. Although only a single TCP meta‐atom is designed for the acoustic metasurface, the geometric phase in each TCP could be adjusted by twisting the PGM‐2, enabling the tunable phase profile for manipulating acoustic waves at will. For example, when these 18 PGMs‐2 are twisted with appropriate orientations to achieve the geometric phase distribution of “0, π/3, 2π/3, π,− π/3, −2π/3” across a period of *p*  =  2λ (six cells cover a complete phase range of 2π), as seen from the phase profile in the left plot of Figure 3d, it enables a phase gradient ξ  =  0.5*k*_0_ to produce the beam steering effect. As predicted by the generalized Snell law,^[^ ^6^, ^7^ ^]^ a high‐efficiency outgoing beam with θ  = sin ^−1^ξ/*k*_0_ = 30°  is demonstrated from both simulated and measured field patterns in the right plot of Figure 3d. The beam splitting effect can be realized by twisting 18 PGMs‐2 with the geometric phase distribution of “0, 0, π, π” across a period of *p*  =  4λ/3 (see the left plot of Figure 3e). The simulated and measured results on the right side of Figure 3e confirm this beam splitting effect^[^ ^42^ ^]^ from two outgoing beams with θ  = sin ^−1^λ/*p* ≈ 49° . In experiments, we utilize a measured region that spans two wavelengths, as seen from the measured field patterns of beam steering and splitting in the region from *z*  =  2.3λ to 4.3λ. Although this measured region is smaller than that in simulations, it is sufficient for observing the tunable wave control effects. Besides, a beam focusing effect is realized by modulating the required geometric phase profile to obtain a focusing length of 4λ, as demonstrated in Figure 3f. To reveal the beam focusing with a spot at *z*  =  5.3λ, the corresponding measured area is shifted to the region from *z*  =  4.3λ to 6.3λ. By observing these simulated results in Figure 3, there are tiny reflections in the incident area (*z* < 0), revealing these tunable wave manipulations with high‐efficiency performance. Due to inevitable acoustic losses and fabrication errors, the transmission fields in experiments are slightly lower than those in simulations. Other tunable wave phenomena [see Text S6, Supporting Information] could be also realized in this acoustic metasurface designed with geometric phases, exhibiting further its powerful ability in controlling acoustic waves at subwavelength scales.

**Figure 4:**
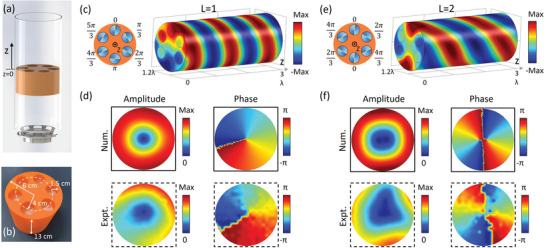
Tunable OAM generation. a) Experimental setup. b) Fabricated sample made of six TCP structures. c,e) are the simulated pressure field patterns of the transmitted vortices with OAM of 1 and 2 by twisting six PGMs‐2 with the geometric phase profile of “0, π/3, 2π/3, π,− π/3, and −2π/3“ and “0, 2π/3, − π/3, 0, 2π/3, −π/3” in the TCP structures, respectively. d,f) are the normalized simulated and measured amplitude and phase distributions on the surface of *z*  =  2λ in (c,e).

Geometric phase in the twisted TCP can be not only used to realize diverse wave functions for acoustic plane waves but also to achieve the tunable generation of acoustic OAM. The tunable OAM generator is critical for acoustic multiplexing OAM communications.^[^ ^29^ ^]^ Here, we design a tunable vortex generator in a tube with a radius of 6 cm and the incident plane wave is generated by a speaker in experiments (see **Figure** **4a**). The OAM generator (see the sample in Figure 4b) is made of 6 TCP structures, which are uniformly arranged on a circle with a radius of 4 cm in a rigid cylinder (radius 6 cm and thickness 13 cm). When these six PGMs‐2 in the OAM generator are twisted with the geometric phase profile of “0, π/3, 2π/3, π,− π/3, and −2π/3”, a high‐efficiency (84.0%) acoustic vortex with OAM of 1 is generated from the incident plane wave in the waveguide, as seen from the simulated transmitted field pattern in Figure 4c. Both simulated and measured amplitude and phase distributions on the surface of *z*  =  2λ reveal the key feature of such an outgoing vortex field (see Figure 4d). When six PGMs‐2 are twisted with the geometric phase profile of “0, 2π/3, − π/3, 0, 2π/3, −π/3”, the high‐efficiency (84.3%) vortex with OAM of 2 is found from the simulated transmitted field pattern in Figure 4e. This transmitted acoustic vortex is also identified by both simulated and measured results of the amplitude and phase information on the surface of *z*  =  2λ (see Figure 4f). Based on the interference method of plane waves, the topological charges of the transmitted vortices are also indicated by the corresponding interferograms (see Figure S9, Supporting Information). Due to inevitable losses and fabrication errors, these phase shifts through TCPs in experiments have some deviations from ideal values, and thus the experimentally measured vortices are not as good as those in simulations. Nevertheless, tunable OAM generation through twisted TCPs is demonstrated in experiments.

## 3 Conclusions

In conclusion, we have demonstrated theoretically and experimentally a new approach for achieving geometric phase by introducing the concept of TCP. Unlike Pancharatnam‐Berry geometric phase relying on the spin‐orbit coupling, this geometric phase physically originates from the cyclic evolution of OAM transformation in mode space, and it is a general principle for classical wave systems, which could offer great opportunities for exploiting promising applications in various wave systems. In particular, the proposed geometric phase paves a new path for controlling scalar fields without intrinsic spin. By applying the concept of TCP in acoustics, we show that the geometric phase offers a simple and robust way to control phase shifts at subwavelength scales, enabling tunable manipulations of both acoustic plane waves and OAM beams. Although these phase gradient cells made of space‐coiling design are designed at the targeted frequency, they can allow the twisted TCP with good performance to work within a wide bandwidth. As a result, the tunable wave control through the TCP‐based metasurface is realized in a wide band of frequencies (see Text S8, Supporting Information). Considering that the TCP structures could be fabricated using natural materials or metamaterials, this geometric phase could be further extended to underwater acoustics,^[^ ^43^, ^44^ ^]^ which may hold significant potential for developing various applications in underwater environments. As the proposed geometric phase can avoid the spin‐orbit coupling process, it could release complex system requirements for realizing some spin‐orbit coupling effects in a more compact and integrated system, which holds significant potential for exploiting geometric phase (e.g., higher‐order geometric phase^[^ ^32^, ^33^ ^]^) and promising applications in various optical systems. For example, this geometric phase might be extended to the system of optical fibers,^[^ ^45^ ^]^ which could offer a new route for controlling light‐matter interactions and designing integrated multifunctional all‐fiber devices. Our work provides a promising foundation for exploring geometric phase in classical wave systems, which offers new possibilities for tailoring wave properties and designing innovative devices with advanced functionalities.

## 4 Experimental Section

### Numerical Simulations

All simulations were performed using COMSOL Multiphysics Pressure Acoustics module. In Figure 3, simulations were carried out in a 2D waveguide with rigid boundaries on the upper and lower surfaces, and the scattering boundary conditions were used for the four sides to avoid undesired reflections. The Gaussian beam is incident on the acoustic metasurface consisting of 18 TCP structures. In Figure 4, this simulation was carried out in a cylindrical waveguide. Both ports of the cylindrical waveguide were set as scattering boundary conditions, the plane wave is incident on the OAM generator consisting of 6 TCP structures.

### Experimental Setup

The experimental samples were fabricated with fused deposition modeling in 3D printing and the printed material was epoxy resin with a density of ≈1160 *kg* *m* ^−3^ and speed of sound ≈2540 *m* *s* ^−1^. As the characteristic impedance of the material is much larger than that of air, the space‐coiling walls can be considered acoustically rigid. In Figure 3, the Gaussian beam in experiments was generated by a loudspeaker array made of 20 speakers, and the transmitted field was scanned using a moving microphone with a step of 5 mm. In Figure 4, the incident plane wave was generated by a loudspeaker with a radius of 5.5 cm and the transmitted field was scanned by a moving microphone with a step of 5 mm.

## Conflict of Interest

The authors declare no conflict of interest.

## Supporting information

Supporting Information

## Data Availability

The data that support the findings of this study are available from the corresponding author upon reasonable request.
