# Supplementary material for: Geometric Phase in Twisted Topological Complementary Pair
Source: Adv Sci (Weinh). 2023 Sep 22;10(33):2304992. doi: 10.1002/advs.202304992 (PMC10667850; doi:10.1002/advs.202304992)
Supplement: Supplementary file 1 — Supporting Information [file ADVS-10-2304992-s001.pdf]

ADVANCED SCIENCE
Open Access

Supporting Information

for *Adv. Sci.*, DOI 10.1002/advs.202304992

Geometric Phase in Twisted Topological Complementary Pair

Kun Zhang, Xiao Li, Daxing Dong, Ming Xue, Wen-Long You, Youwen Liu, Lei Gao, Jian-Hua Jiang, Huanyang Chen, Yadong Xu* and Yangyang Fu**

Supplementary information for

Geometric Phase in Twisted Topological Complementary Pair

Kun Zhang[1,2,6], Xiao Li[1,2,6], Daxing Dong[1,2,6], Ming Xue[1,2], Wen-Long You[1,2], Youwen Liu[1,2], Lei Gao[3], Jian-Hua Jiang[3], Huanyang Chen[4*], Yadong Xu[3†], and Yangyang Fu[1,2,5‡]

[1]*College of Physics, Nanjing University of Aeronautics and Astronautics, Nanjing 211106, China*
[2]*Key Laboratory of Aerospace Information Materials and Physics (NUAA), MIIT, Nanjing 211106, China*
[3]*School of Physical Science and Technology & Jiangsu Key Laboratory of Thin Films, Soochow University, Suzhou 215006, China.*
[4]*Department of Physics, Xiamen University, Xiamen, 361005, China*
[5]*State Key Laboratory of Mechanics and Control for Aerospace Structures, Nanjing University of Aeronautics and Astronautics, Nanjing 210016, China*
[6]*The authors contributed equally to this work*

Text

1. Numerical demonstration for geometric phase using acoustic waves and linearly polarized light....... 2
2. Coupled mode theory for geometric phase in a subwavelength acoustic waveguide. 4
3. Analytical and numerical demonstrations for acoustic geometric phase. 8
4. Design for TCP 9
5. Experimental setup 11
6. Simulated field patterns of tunable transmission and asymmetric transmission 12
7. The interferograms of the generating vortices with plane waves 14
8. The spectrum performance of twisted TCPs and the tunable wave control 15
Reference 17

* kenyon@xmu.edu.cn
† ydxu@suda.edu.cn
‡ yyfu@nuaa.edu.cn

1. Numerical demonstration for geometric phase using acoustic waves and linearly polarized light

As the geometric phase proposed in this work results from the cyclic evolution of orbital-angular-momentum (OAM) transformation in mode space, it can work in these wave systems without relying on intrinsic spin. Due to the Rayleigh diffraction effect [1], a large-scale topological complementary pair (TCP) a diameter of $D = 4\lambda$, where λ is the wavelength in free space, is used to demonstrate this geometric phase for acoustic waves and linearly polarized light in free space. We employ a pair of gradient index plates (GIPs) made of gradient index media (see the inset in **Figure S1a**) to implement the TCP in acoustic waves at a specific wavelength of $\lambda = 10$ cm. The gradient media of $n_j = 1 + (j-1)\lambda/mh$ $(j = 1, 2 \cdots m)$ in GIPs are considered and m is the number of the discrete plates for designing intrinsic topological charge (ITC) within the structure. These gradient index design in acoustics could be easily realized by soft graded-porous silicone rubber [2] in water. Moreover, a pair of spiral phase plates (SPPs) (see inset in Figure S1b) made by silicon dioxide with refractive index $n = 1.5$ is utilized to design the TCP. A linearly-polarized light at a wavelength of $\lambda = 500$ nm is used to observe the geometric phase. We study three cases by designing these TCPs with q=1, 2, 3, respectively.

By placing the designed TCP in the center of free space with dimensions of $11\lambda \times 6\lambda \times 6\lambda,$ we carry out numerical simulations of COMSOL to reveal the geometric phase. To minimize undesired reflections, the simulation region is enveloped by perfectly matched layers with a thickness of 0.5λ and the incident wave used in the simulations is modeled as a Gaussian beam with a beam radius of 2λ. We examine the phase and transmission of the transmitted waves at 3λ away from the TCP. By twisting the TCP, the corresponding phase and transmission versus the twist angle are depicted in Figure S1. For these TCPs with q=1, 2, 3, the resulting transmitted phases of the output fields can exhibit a linearly-varying phase shift of $\varphi_g = \theta$, $\varphi_g = 2\theta$ and $\varphi_g = 3\theta$, respectively. The transmission efficiency falls short of unity due to undesirable reflections and the presence of an unstable nearly-wavelength OAM mode. By observing these results in Figure S1, we find that the whole transmission amplitude in both cases gradually decreases with the increase of q, which is mainly caused by the enlarged inhomogeneous property of gradient index materials or gradient thickness design in TCPs that can lead more reflection. In addition, twisting the TCP can lead to stronger wave interference due to the enlarged inhomogeneous property, so that the transmission curve can fluctuate up and down stronger. Consequently, numerical evidence is presented to demonstrate the generality of the proposed geometric phase for acoustic waves

and linearly polarized light in free space.

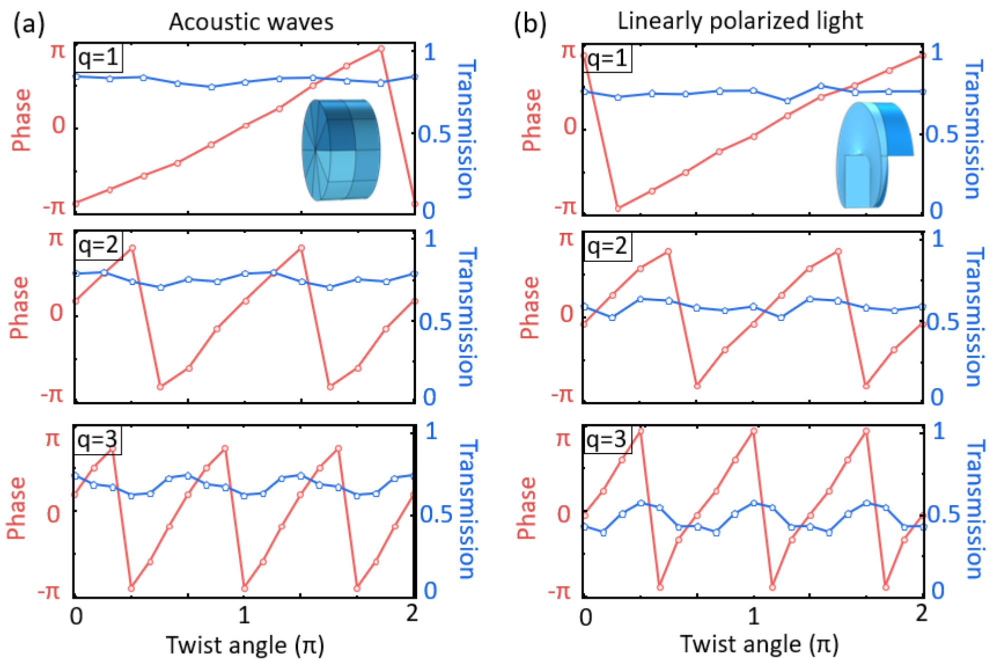

Figure S1. Numerical demonstrations for generation of geometric phase in free space. The transmission and phase profiles of the TCPs with q=1, 2, 3 versus the twist angle for acoustic waves (a) and linearly polarized light (b).

2. Coupled mode theory for geometric phase in a subwavelength acoustic waveguide.

To demonstrate the geometric phase enabled by the subwavelength TCP in the waveguide, we employ the coupled mode theory (CMT) to conduct an analytical study. In this cylindrical waveguide system (r,ϕ,z), the acoustic pressure fields inside it are composed of these guided (vortex) modes,

$$p = A^{l,\alpha} J_l(k_r^{l,\alpha} r)\exp(ik_z^{l,\alpha} z + il\phi)\,, \tag{S1}$$

where l is the topological charge, $k_z^{l,\alpha}$ and $k_r^{l,\alpha}$ are the transverse and longitudinal wave numbers, respectively, which meet $(k_r^{l,\alpha})^2 + (k_z^{l,\alpha})^2 = k_0^2$ ($k_0 = 2\pi/\lambda$ is the wavevector in free space), $J_l(k_r^{l,\alpha} r)$ is the *l*-th Bessel function and $k_r^{l,\alpha} R$ is the solution of the equation $\frac{\partial J_l(k_r^{l,\alpha} r)}{\partial (k_r^{l,\alpha} r)}|_{r=R} = 0$. We consider that the fundamental plane wave is incident on the PGM-1 from the left side, and the total acoustic pressure fields in region I (see **Figure S2a**) are composed of the incident plane wave and these reflected guided modes,

$$p^{\mathrm{I}} = \sum_{l,\alpha}[\delta_{l,0}\delta_{\alpha,0}\exp(ik_z^{l,\alpha} z) + \Re^{l,\alpha}\exp(-ik_z^{l,\alpha} z)]J_l(k_r^{l,\alpha} r)\exp(il\phi)\,, \tag{S2}$$

where $\Re^{l,\alpha}$ is the reflection coefficient of the corresponding guided mode.

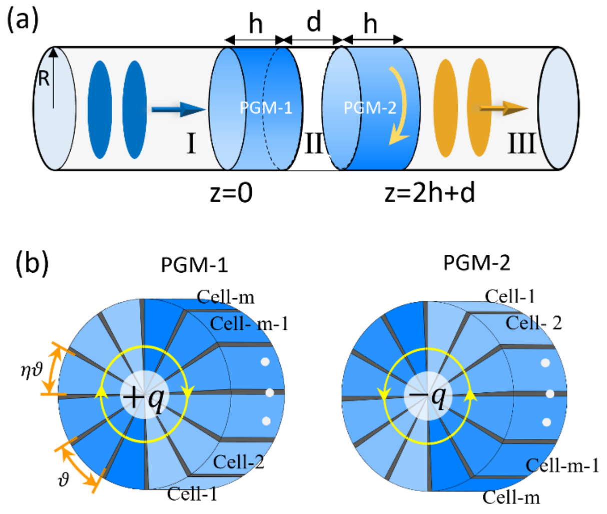

Figure S2. The analytical model of the TCP (PGM-1 and PGM-2) in a subwavelength cylindrical waveguide, in which an extremely tiny air gap ($d \to 0$) between PGM-1 and PGM-2 is introduced to match the boundary conditions at the interface of PGMs.

In order to match the boundary conditions at the interface between the PGM-1 and the PGM-2, an extremely tiny air gap ($d \to 0$) is introduced in the analytical model, and the acoustic fields inside it (region II in Figure S2a) consist of forward and backward guided modes,

$$p^{\mathrm{II}} = \sum_{l,\alpha} [C^{l,\alpha} \exp(ik_z^{l,\alpha} z) + D^{l,\alpha} \exp(-ik_z^{l,\alpha} z)] J_l(k_r^{l,\alpha} r) \exp(il\phi), \tag{S3}$$

where $C^{l,\alpha}$ and $D^{l,\alpha}$ are the complex amplitudes of forward and backward guided modes. In the transmitted side (region III), the transmitted waves could be written as the superposition of guided modes,

$$p^{\mathrm{III}} = \sum_{l,\alpha} \Im^{l,\alpha} J_l(k_r^{l,\alpha} r) \exp(il\phi) \exp(ik_z^{l,\alpha} z), \tag{S4}$$

where $\Im^{l,\alpha}$ is the transmission coefficient of the corresponding guided mode.

As each PGM with thickness h is composed of q groups of fanlike supercells and the supercell is designed by m groups of fanlike cells with angular width $\vartheta = 2\pi / (mq)$, leading to mq groups of cells in total. Each cell is composed of the impedance-matched material with angular width $\eta\vartheta$ and the sound-hard medium with angular width $(1-\eta)\vartheta$, where η is the filling ratio. The central angular position of the *j*-th cell in the PGM-1 is $\phi_j^1 = \vartheta / 2 + (j-1)\vartheta$, $j = 1, 2, 3 ... mq$ and it is $\phi_j^2 = \vartheta / 2 + (1-j)\vartheta + \theta$, $j = 1, 2, 3 ... mq$ in the PGM-2 (θ is the twist angle of the PGM-2). The impedance-matched material in the *j*-th cell of mq cells is,

$$\rho_j / \rho_0 = n_j = \begin{cases} 1 + (j-1)\lambda / (mh), & j = 1 : m \\ 1 + (j-1-m)\lambda / (mh), & j = (m+1) : 2m \\ \cdots & \\ 1 + (j-1-(l^{\xi}-1)m)\lambda / (mh), & j = ((q-1)m+1) : mq \end{cases} \quad (j = 1, 2, 3 ... mq). \tag{S5}$$

Due to the subwavelength feature of the PGMs, we only consider the fundamental mode in these cells and the propagation constant is $\beta_j = n_j k_0$. As a result, the acoustic pressure field in the *j*-th cell of the PGM-1 can be written as,

$$P_j^1 = \begin{cases} A_j \exp(i\beta_j z) + B_j \exp(-i\beta_j z), & \phi \in [\phi_j^1 - \eta\vartheta / 2, \phi_j^1 + \eta\vartheta / 2] \\ 0, & \phi \in [\phi_j^1 - \vartheta / 2, \phi_j^1 - \eta\vartheta / 2) \,\&\, (\phi_j^1 + \eta\vartheta / 2, \phi_j^1 + \vartheta / 2] \end{cases}, \tag{S6}$$

where A_j and B_j are the complex amplitudes of forward and backward plane waves. In the PGM-2 region, the pressure field in the j -th cell is,

$$P_j^2 = \begin{cases} E_j \exp(i\beta_j z) + F_j \exp(-i\beta_j z), & \phi \in [\phi_j^2 - \eta\vartheta / 2, \phi_j^2 + \eta\vartheta / 2] \\ 0, & \phi \in [\phi_j^2 - \vartheta / 2, \phi_j^2 - \eta\vartheta / 2) \,\&\, (\phi_j^2 + \eta\vartheta / 2, \phi_j^2 + \vartheta / 2] \end{cases}, \tag{S7}$$

where E_j and F_j are the complex amplitudes of forward and backward plane waves. The velocity fields in these regions are obtained from $v_z = -\frac{1}{i\omega\rho}\frac{\partial p}{\partial z}$, i.e.,

$$v_z^{\mathrm{I}} = -\frac{1}{\omega\rho_0}\sum_{l,\alpha}[\delta_{l,0}\delta_{\alpha,0}\exp(ik_z^{l,\alpha}z) - \Re^{l,\alpha}\exp(-ik_z^{l,\alpha}z)]k_z^{l,\alpha}J_l(k_r^{l,\alpha}r)\exp(il\phi), \tag{S8}$$

$$v_z^{\mathrm{II}} = -\frac{1}{\omega\rho_0}\sum_{l,\alpha}[C^{l,\alpha}\exp(ik_z^{l,\alpha}z) - D^{l,\alpha}\exp(-ik_z^{l,\alpha}z)]k_z^{l,\alpha}J_l(k_r^{l,\alpha}r)\exp(il\phi), \tag{S9}$$

$$v_z^{\mathrm{III}} = -\frac{1}{\omega\rho_0}\sum_{l,\alpha}\Im^{l,\alpha}k_z^{l,\alpha}J_l(k_r^{l,\alpha}r)\exp(il\phi)\exp(ik_z^{l,\alpha}z), \tag{S10}$$

$$v_{z,j}^1 = \begin{cases} -\frac{\beta_j}{\omega\rho_j}[A_j\exp(i\beta_j z) - B_j\exp(-i\beta_j z)], & \phi\in[\phi_{1,j}-\eta\vartheta/2, \phi_{1,j}+\eta\vartheta/2] \\ 0, & \phi\in[\phi_{1,j}-\vartheta/2, \phi_{1,j}-\eta\vartheta/2)\,\&\,(\phi_{1,j}+\eta\vartheta/2, \phi_{1,j}+\vartheta/2] \end{cases}, \tag{S11}$$

$$v_{z,j}^2 = \begin{cases} -\frac{\beta_j}{\omega\rho_j}[E_j\exp(i\beta_j z) - F_j\exp(-i\beta_j z)], & \phi\in[\phi_{2,j}-\eta\vartheta/2, \phi_{2,j}+\eta\vartheta/2] \\ 0, & \phi\in[\phi_{2,j}-\vartheta/2, \phi_{2,j}-\eta\vartheta/2)\,\&\,(\phi_{2,j}+\eta\vartheta/2, \phi_{2,j}+\vartheta/2] \end{cases}. \tag{S12}$$

We apply the continuous boundary condition of the pressure field at $z=0$ via Eq. (S2) and Eq. (S6) by performing the integral in each cell with $\phi_j^1 - \eta\vartheta/2 \le \phi \le \phi_j^1 + \eta\vartheta/2$ and $0 \le r \le R$, we can obtain

$$\sum_{l,\alpha}[\delta_{l,0}\delta_{\alpha,0} + \Re^{l,\alpha}]\int_0^R J_l(k_r^{l,\alpha}r)r\mathrm{d}r\int_{\phi_j^1-\eta\vartheta/2}^{\phi_j^1+\eta\vartheta/2}\exp(il\phi)\mathrm{d}\phi = [A_j + B_j]\int_0^R r\mathrm{d}r\int_{\phi_j^1-\eta\vartheta/2}^{\phi_j^1+\eta\vartheta/2}\mathrm{d}\phi, \tag{S13}$$

which is further simplified to,

$$\sum_{l,\alpha}[\delta_{l,0}\delta_{\alpha,0} + \Re^{l,\alpha}]\exp(il\phi_j^1)\mathrm{sinc}(\frac{1}{2}l\eta\vartheta)\int_0^R J_l(k_r^{l,\alpha}r)r\mathrm{d}r = \frac{1}{2}R^2[A_j + B_j]. \tag{S14}$$

Likewise, by applying continuous boundary condition of the pressure field at $z=h$, $z=h+d$ and $z=2h+d$, we have

$$\begin{aligned}&\sum_{l,\alpha}[C^{l,\alpha}\exp(ik_z^{l,\alpha}h) + D^{l,\alpha}\exp(-ik_z^{l,\alpha}h)]\exp(il\phi_j^1)\mathrm{sinc}(\frac{1}{2}l\eta\vartheta)\int_0^R J_l(k_r^{l,\alpha}r)r\mathrm{d}r \\ &= \frac{1}{2}R^2[A_j\exp(i\beta_j h) + B_j\exp(-i\beta_j h)],\end{aligned} \tag{S15}$$

$$\begin{aligned}&\sum_{l,\alpha}[C^{l,\alpha}\exp(ik_z^{l,\alpha}(h+d)) + D^{l,\alpha}\exp(-ik_z^{l,\alpha}(h+d))]\exp(il\phi_j^2)\mathrm{sinc}(\frac{1}{2}l\eta\vartheta)\int_0^R J_l(k_r^{l,\alpha}r)r\mathrm{d}r \\ &= \frac{1}{2}R^2[E_j\exp(i\beta_j(h+d)) + F_j\exp(-i\beta_j(h+d))],\end{aligned} \tag{S16}$$

$$\begin{aligned}&\sum_{l,\alpha}\Im^{l,\alpha}\exp(ik_z^{l,\alpha}(2h+d))\exp(il\phi_j^2)\mathrm{sinc}(\frac{1}{2}l\eta\vartheta)\int_0^R J_l(k_r^{l,\alpha}r)r\mathrm{d}r \\ &= \frac{1}{2}R^2[E_j\exp(i\beta_j(2h+d)) + F_j\exp(-i\beta_j(2h+d))].\end{aligned} \tag{S17}$$

The continuous boundary condition of the total velocity fields is considered at the interface $z=0$ and the surface integral is operated for in whole interface ($0 \le \phi \le 2\pi$ and $0 \le r \le R$) by multiplying both

Eq. (S8) and Eq. (S11) with $[J_l(k_r^{l,\alpha}r)\exp(il\varphi)]^*$, then we have

$$\begin{aligned}&\frac{2\pi}{\eta\vartheta\rho_0}[\delta_{l,0}\delta_{\alpha,0}-\Re^{l,\alpha}]k_z^{l,\alpha}\int_0^R[J_l(k_r^{l,\alpha}r)]^2 r\mathrm{d}r\\&=\sum_j\frac{\beta_j}{\rho_j}[A_j-B_j]\exp(-il\phi_j^1)\mathrm{sinc}(\frac{1}{2}l\eta\vartheta)\int_0^R J_l(k_r^{l,\alpha}r)r\mathrm{d}r.\end{aligned}\tag{S18}$$

Similarly, we can get the following equations by applying the continuous boundary condition of total velocity fields at $z=h$, $z=h+d$ and $z=2h+d$,

$$\begin{aligned}&\frac{2\pi}{\eta\vartheta\rho_0}[C^{l,\alpha}\exp(ik_z^{l,\alpha}h)-D^{l,\alpha}\exp(-ik_z^{l,\alpha}h)]k_z^{l,\alpha}\int_0^R[J_l(k_r^{l,\alpha}r)]^2 r\mathrm{d}r\\&=\sum_j\frac{\beta_j}{\rho_j}[A_j\exp(i\beta_jh)-B_j\exp(-i\beta_jh)]\exp(-il\phi_j^1)\mathrm{sinc}(\frac{1}{2}l\eta\vartheta)\int_0^R J_l(k_r^{l,\alpha}r)r\mathrm{d}r,\end{aligned}\tag{S19}$$

$$\begin{aligned}&\frac{2\pi}{\eta\vartheta\rho_0}[C^{l,\alpha}\exp(ik_z^{l,\alpha}(h+d))-D^{l,\alpha}\exp(-ik_z^{l,\alpha}(h+d))]k_z^{l,\alpha}\int_0^R[J_l(k_r^{l,\alpha}r)]^2 r\mathrm{d}r\\&=\sum_j\frac{\beta_j}{\rho_j}[E_j\exp(i\beta_j(h+d))-F_j\exp(-i\beta_j(h+d))]\exp(-il\phi_j^2)\mathrm{sinc}(\frac{1}{2}l\eta\vartheta)\int_0^R J_l(k_r^{l,\alpha}r)r\mathrm{d}r,\end{aligned}\tag{S20}$$

$$\begin{aligned}&\frac{2\pi}{\eta\vartheta\rho_0}\Im^{l,\alpha}\exp(ik_z^{l,\alpha}(2h+d))k_z^{l,\alpha}\int_0^R[J_l(k_r^{l,\alpha}r)]^2 r\mathrm{d}r\\&=\sum_j\frac{\beta_j}{\rho_j}[E_j\exp(i\beta_j(2h+d))-F_j\exp(-i\beta_j(2h+d))]\exp(-il\phi_j^2)\mathrm{sinc}(\frac{1}{2}l\eta\vartheta)\int_0^R J_l(k_r^{l,\alpha}r)r\mathrm{d}r.\end{aligned}\tag{S21}$$

By solving Eq. (S14) through Eq. (S21), we can analytically obtain all the unknown coefficients of $\Re^{l,\alpha}$ and $\Im^{l,\alpha}$. Owing to the feature of the subwavelength waveguide, only plane wave mode ($l=0;\alpha=0$) is the propagation mode. Therefore, we can obtain the transmission efficiency and phase shift of the outgoing wave in the waveguide by the complex amplitude of $\Im^{0,0}$.

3. Analytical and numerical demonstrations for acoustic geometric phase.

We use different TCP designs with $q = 1, 2, 3, 4$ to verify the generation of acoustic geometric phase (AGP) of arbitrary order in the proposed waveguide system. For example, the radius of the waveguide is $R = 0.1\lambda$, the PGM thickness is $h = 0.5\lambda$, and the filling ratio is $\eta = 0.9$. **Figure S3** shows both analytical calculations of the coupled mode theory and numerical simulations (COMSOL) for these four cases. In the CMT, we consider an air gap of $d = 0.00001\lambda$ between the PGM-1 and PGM-2 to analytically calculate the transmission efficiency and the corresponding phase shift. By twisting the PGM-2, we can see that the phase shift ($\arg(\mathfrak{I}^{0,0})$) of the outgoing wave with a unity efficiency has a linear dependence on the twist angle of the PGM-2 and it covers the full phase range of 2π in the way of $\varphi_g = q\theta$, which is consistent with Eq. (5) in the main text. To check the CMT results, numerical simulations are also performed by considering the PGMs without gap ($d = 0$). We can see that the resulting phase shift can exhibit a linear dependence on the twist angle in a robust manner, consistent with the theoretical and analytical predictions. In particular, the transmission in all cases has a slight fluctuation with the twist angle, which is consistently revealed by analytical and numerical results. Therefore, the proposed AGP enabled by the subwavelength TCP is demonstrated both analytically and numerically.

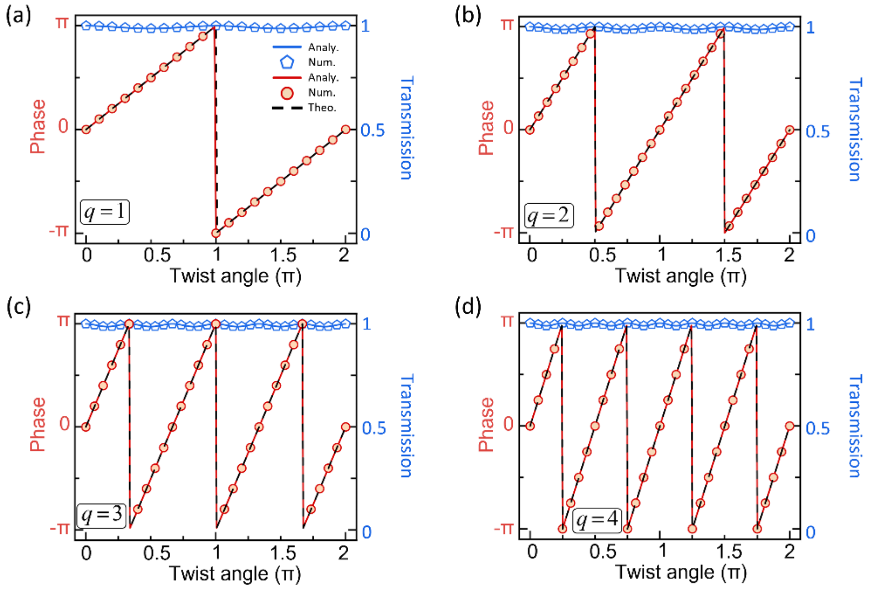

Figure S3. Analytical and numerical demonstrations for robust generation of AGP. The transmission and phase profiles of the TCP versus the twist angle (θ) of PGM-2. (a) $q = 1,\ m = 20$; (b) $q = 2,\ m = 15$; (c) $q = 3,\ m = 10$; (d) $q = 4,\ m = 8$; In plots, the analytical calculations are presented by the solid curves and the numerical simulations are indicated by circles. The theoretical results of $\varphi_g = q\theta$ are shown by the black dashed curves.

4. Design for TCP

To demonstrate the geometric phase in acoustics, we design a PGM with $q=2$ at the wavelength $\lambda=10\text{cm}$. For the convenience of sample fabrication, $R=0.15\lambda$ and $m=6$ are set for the PGM design. In this case, the angular width of the fanlike cells of the PGM is $\vartheta=360^{\circ}/(mq)=30^{\circ}$, and the thickness of the PGM is set with $h=4.5\text{cm}$. The fanlike cell (see **Figure S4a**) is designed by rotating its cross-section (see Figure S4b) along the z axis with $\vartheta=30^{\circ}$. The cross-section structure is designed using the coiling-up space structure, which is made of seven building blocks with the same space symmetrically placed in the waveguide (four blocks on the top and three blocks on the bottom). The thickness of waveguide walls and these building blocks are both $t = 1$ mm. Two sound-hard surfaces with thickness of 0.25 mm are added on either side of each cell to form a fanlike waveguide, as shown in Figure S4a. By changing the height b and the space f of these building blocks, the transmission and phase profiles of the fanlike cell are numerically tested in a fanlike waveguide. Finally, six different fanlike cells with varying geometric sizes (see Table S1) are found to obtain the required phase shifts and nearly unity transmission. As shown in Figure S4c, the realization of a phase difference $\Delta\varphi=\pi/3$ between two adjacent cells and a nearly unity transmission efficiency in each cell is achieved. With these six fanlike cells, the PGM with $q=2$ is designed, see Figure S4d.

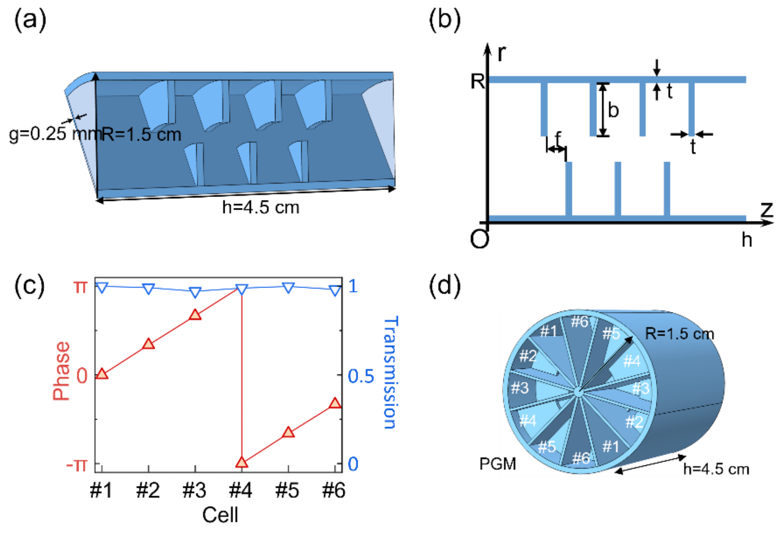

Figure S4. PGM design. (a) Schematic diagram of the designed fanlike cell, which is realized by rotating its azimuthal section. (b) consisting of the coiling-up space structure. (c) Phase and transmission profiles of the designed fanlike cells. (d) Topography of the designed PGM with $q=2$.

Table S1. Geometric parameters of the designed six fanlike cells

cells	#1	#2	#3	#4	#5	#6
n_1	0	4	4	4	4	4
n_2	0	3	3	3	3	3
b/mm	0	5.25	6.75	7.90	9.00	10.10
f/mm	\	2.97	1.77	5.23	3.43	1.57
T	100%	99.24%	97.22%	98.99%	99.94%	98.26%
φ/π	0	0.338	0.667	-0.999	-0.659	-0.331

Further, we use two designed PGMs that spatially placed with opposite helicity to construct a TCP with ITC of $q=2$. For the aim of controlling acoustic waves in a planar geometry, the cylindrical waveguide is wrapped by a square cross-section block with length $\lambda/3=3.33cm$, which is slightly larger than the diameter (3 cm) of the waveguide. Two matching layers (empty waveguides) with length 2 cm are added on both sides of the TCP structure to obtain the AGP with perfect efficiency, see **Figure S5a**. By twisting the PGM-2, the phase shift of the outgoing wave through the designed TCP has a linear dependence of $\varphi_g = 2\theta$ and its transmission efficiency is nearly unity, as shown in Figure S5b, which is consistent with both theoretical and analytical results.

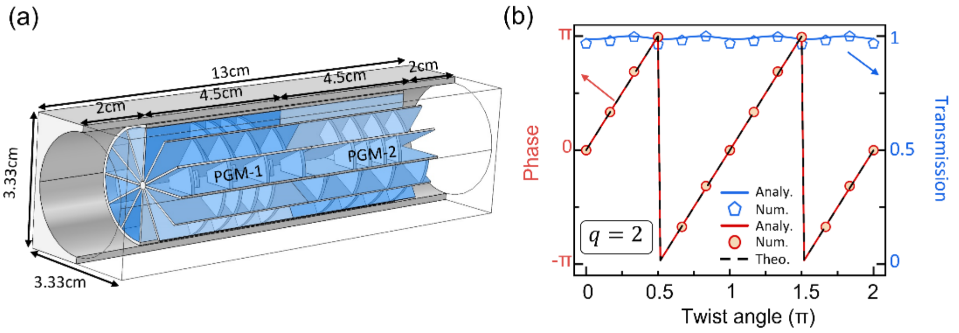

Figure S5. (a) Structural diagram of the designed TCP. (b) The numerical transmission and phase profiles (symbols) of the designed TCP, consistent with theoretical (dashed curve) and analytical results (solid curves).

5. Experimental setup

Experiments are performed in a two-dimensional acoustic waveguide realized by two parallel acrylics plates with a separation of 3.33 cm (see **Figure S6**), which can support the fundamental guided mode like plane wave in free space at the operating frequency 3430 Hz (a wavelength 10 cm). The sample made of 18 TCP meta-atoms is fabricated by 3D printing with commercial photopolymer materials, which is considered as acoustically rigid owing to its characteristic impedance larger than that of air. A computer-controlled speaker array, operated by a PCIe card (PCIe 9310, ART Technology), is utilized to produce a normally incident gaussian beam. To avoid the undesired reflection on the boundaries of the waveguides, foams are placed to absorb the incoming waves. The acoustic pressure signal is captured by a mobile microphone (BSWA MPA436A, BSWA Technology) with a step of 0.5 cm and transmitted to a computer via a USB data acquisition card (VK 710H, VKinging Electronics) for analysis and processing.

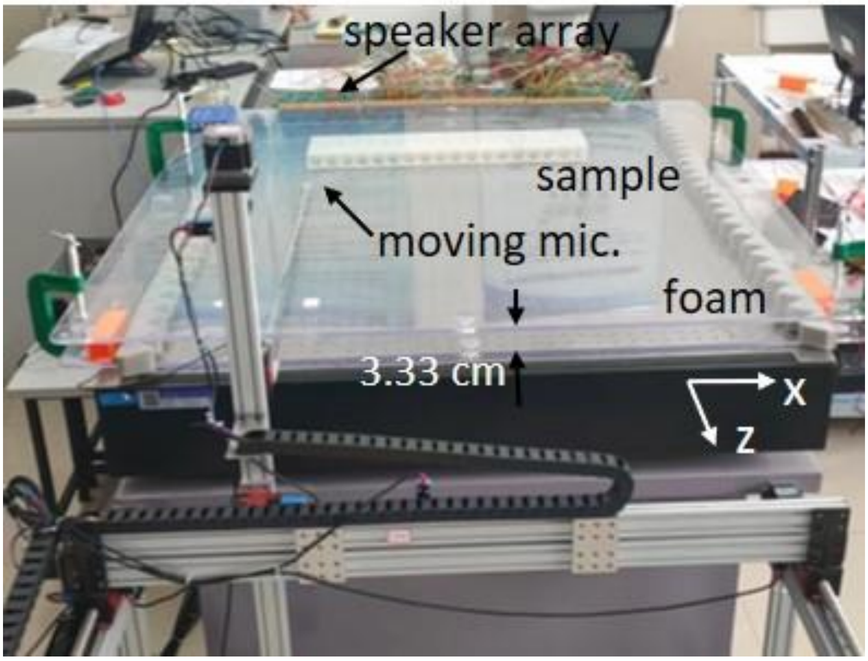

Figure S6. Photograph of Experimental setup.

6. Simulated field patterns of tunable transmission and asymmetric transmission

In addition to these wave functions revealed in the main text, other wave effects, such as tunable transmission and asymmetric transmission could be also realized in the TCP-based acoustic metasurface (1D array made of 18 fabricated TCP structures). By twisting 18 PGM-2 with the identical orientation to obtain the same phase shift, owing to the perfect transmission of the TCP, total transmission is realized, as seen from the simulated field in **Figure S7a**. However, by twisting 18 PGMs-2 with the geometric phase distribution of "0, π" cross a period of $p = 2\lambda/3$, leading to a phase gradient $\xi = 1.5k_0$, total reflection can occur owing to the binary design [3], as shown in Figure S7b.

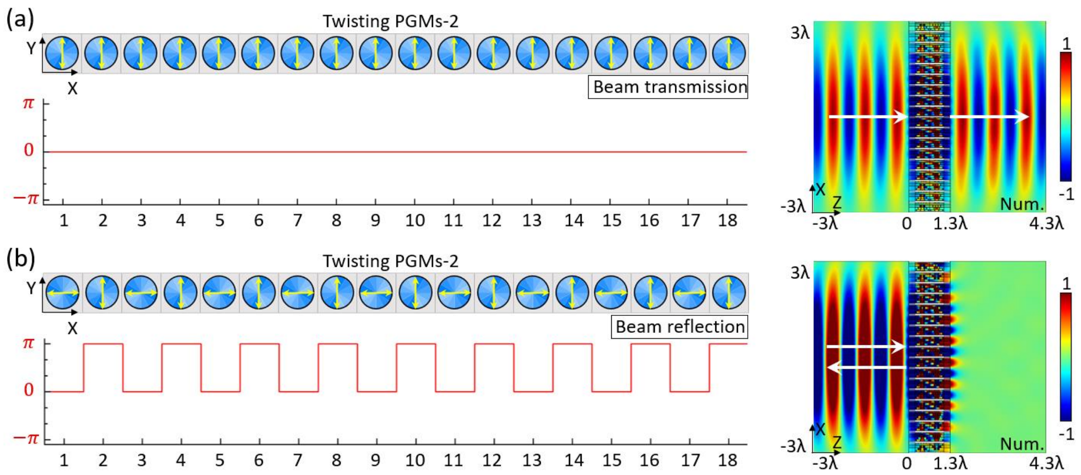

Figure S7. Numerical demonstrations for the tunable acoustic metasurface with total reflection (a) and total transmission (b) effects by twisting 18 PGMs-2 with different orientations (yellow arrows) to achieve the required geometric phase profiles (red lines), respectively.

In addition, the acoustic metasurface also can achieve asymmetric transmission by twisting 18 PGMs-2 with the geometric phase distribution of "0, $2\pi/3, -2\pi/3$" cross a period of $p = \lambda$, which can generate a phase gradient $\xi = 1.0k_0$. For the incident beam from opposite side, which can take different diffraction orders of n=1 and n=-1, asymmetric transmission can happen arising from the parity-dependent diffraction law [4]. For example, as shown in **Figure S8**, when the incident gaussian beam with $\theta = 30°$ bumps on the metasurface, high-efficiency negative refraction happens, which is observed from the simulated field pattern. When the gaussian beam with $\theta = 30°$ is incident on the metasurface from the opposite side, high-efficiency retroreflection occurs, leading to a low-efficiency negative refraction (transmission).

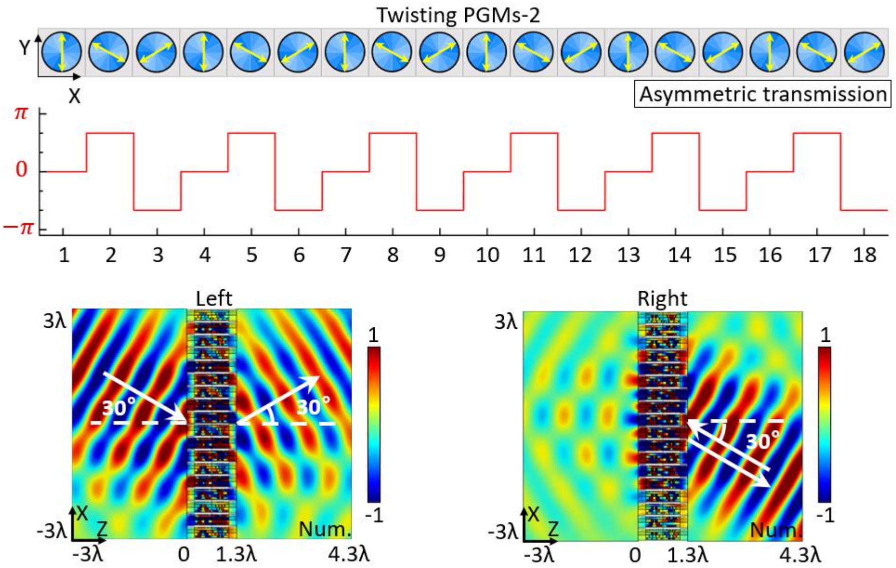

Figure S8. Numerical demonstrations for the tunable acoustic metasurface with asymmetric transmission by twisting 18 PGMs-2 with different orientations (yellow arrows) to achieve the required AGP profiles (red lines).

7. The interferograms of the generating vortices with plane waves

The interferograms of plane waves is an alternative approach for revealing the topological charge carried by vortex beams, which is widely used in optics [5]. By examining the simulated fields of the transmitted vortices within the waveguide (Figure 4 in the main text), we presented the corresponding interferograms in **Figure S9**. These interferograms manifest the topological charges of the transmitted vortices through the number of phase singularities or null amplitude. For instance, an interferogram generated by a planar guided mode ($l = 0$) and a vortex with a topological charge of $l = 1$ exhibits a single point of phase singularity (see Figure S9a), whereas the interferogram for a vortex with a topological charge of $l = 2$ displays two such points (see Figure S9b). As these vortex modes exist in the waveguide system, the corresponding interferograms are different than these in free space. Nevertheless, this method provides a straightforward means of verifying the topological charge carried by a vortex.

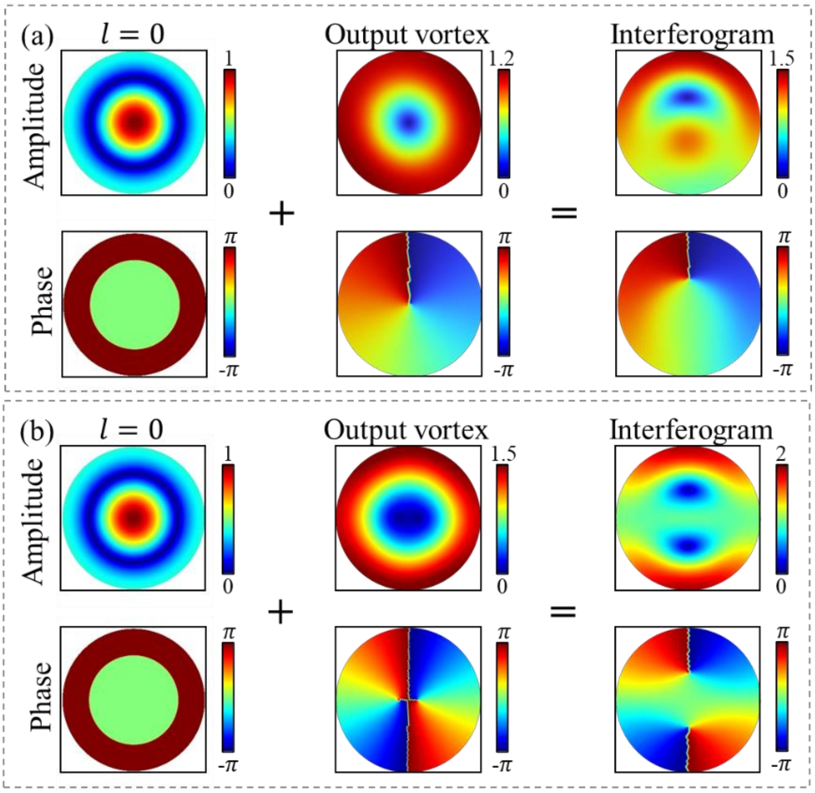

Figure S9. Interferograms generated from the planar guided mode and the simulated ouput vortices through six twisted TCPs, where (a) and (b) are for L=1 and L=2 cases in Fig. 4.

8. The spectrum performance of twisted TCPs and the tunable wave control

In the main text, we have demonstrated tunable wave control enabled by the TCP-based acoustic metasurface at the targeted frequency of 3430 Hz. Although phase gradient cells consisting of space-coiling structures are specifically designed at the targeted one, the necessary phase and transmission profiles for the twisted TCP can still be achieved when the operating frequency deviates from the targeted one. As a result, the TCP can still exhibit excellent performance within a wide frequency range, leading to a broadband performance of the tunable wave control. Here, we conducted numerical investigations on the phase shift and transmission characteristics of the twisted TCP at six different angles, encompassing various operating frequencies. The results, presented in **Figure S10**, reveal that the phase shift remains linear, covering a 2π phase, while exhibiting high transmission within the frequency band of 3.1 to 3.9 kHz. Specific frequency points display significantly reduced transmission (as observed in the black zone in Figure S10a), thereby impacting the corresponding phase shift. To further validate our findings, we give the simulated the field patterns to illustrate the tunable wave effects of beam steering, splitting, and focusing at different frequencies spanning from 3.1 to 3.9 kHz, with a step of 100 Hz. These simulations, depicted in **Figure S11**, **S12**, and **S13**, demonstrate outstanding performance for the selected frequencies across all three effects. Notably, the operating frequency band of 3.43 to 3.9 kHz exhibits superior performance compared to the lower band of 3.1 to 3.3 kHz. It can be concluded that the TCP-based metasurface enables tunable wave control over a wide range of frequencies.

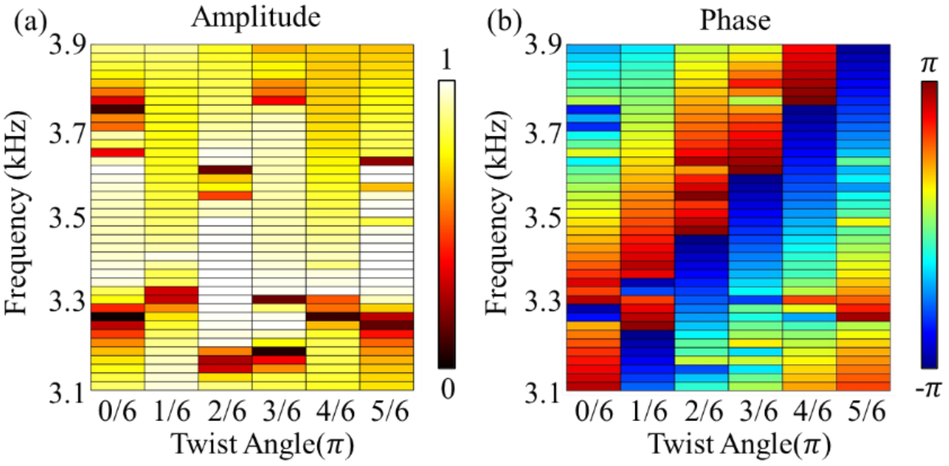

Figure S10. Amplitude (a) and phase (b) profiles of the twisted TCP versus the twist angle and the operating frequency.

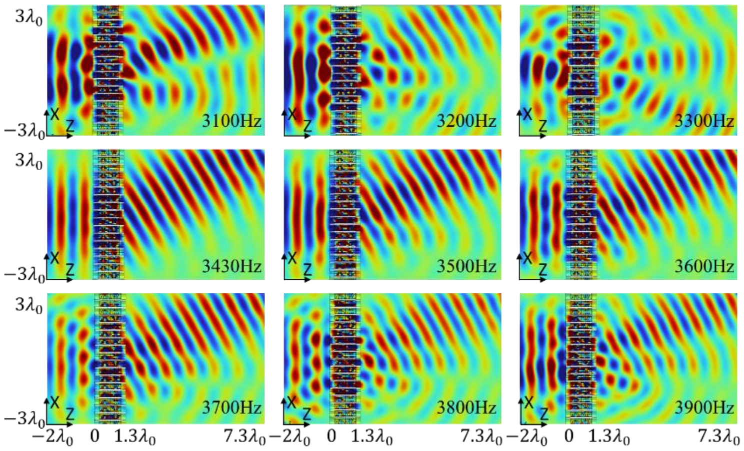

Figure S11. The simulated field patterns of the beam steering at different operating frequencies.

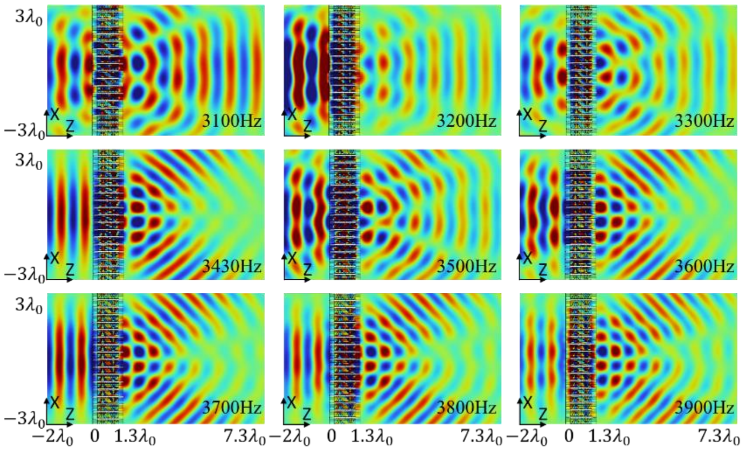

Figure S12. The simulated field patterns of the beam splitting at different operating frequencies.

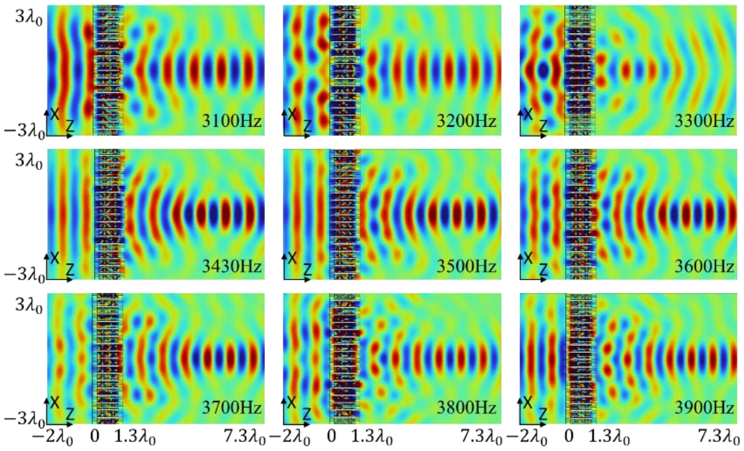

Figure S13. The simulated field patterns of the beam focusing at different operating frequencies.

Reference

1. N. Jiménez, J. Ealo, R. D. Muelas-Hurtado, A. Duclos, V. Romero-García, Subwavelength Acoustic Vortex Beams Using Self-Demodulation. *Phys. Rev. Appl.* 15, 054027 (2021).

2. Y. Jin, R. Kumar, O. Poncelet, O. Mondain-Monval, T. Brunet, Flat acoustics with soft gradient-index metasurfaces. *Nat. Commun.* 10, 143 (2019).

3. Y. Fu, J. Tao, A. Song, Y. Liu, Y. Xu, Controllably asymmetric beam splitting via gap-induced diffraction channel transition in dual-layer binary metagratings. *Front. Phys.* 15, 52502 (2020).

4. Y. Fu, C. Shen, Y. Cao, L. Gao, H. Chen, C. T. Chan, S. A. Cummer, Y. Xu, Reversal of transmission and reflection based on acoustic metagratings with integer parity design. *Nat. Commun.* 10, 2326 (2019).

5. N. Zhou, J. Liu, J. Wang, Reconfgurable and tunable twisted light laser. *Sci. Rep*. 8, 11394 (2018).
